# Which is more biased: Standardized weather stations or microclimatic sensors?

**DOI:** 10.1002/ece3.3965

**Published:** 2018-05-07

**Authors:** Michael B. Ashcroft

**Affiliations:** ^1^ Centre for Sustainable Ecosystem Solutions School of Biological Sciences University of Wollongong Wollongong NSW Australia

**Keywords:** climate loggers, microclimate, observation error, radiation shields, standardized observations, temperature sensors

## Abstract

**Linked Article**: https://doi.org/10.1002/ece3.3972

A recent article in Ecology and Evolution by Terando, Youngsteadt, Meineke, and Prado (2017) compared observations from standardized weather stations (shielded at a height of ~2 m) with those from cheaper microclimatic sensors with custom‐built radiation shields. They found there could be biases of up to 3–5°C under full sun conditions on hot days. They concluded that it is critical to standardize the collection of microclimate data, to reduce biases, and to ensure observations from different studies are comparable. Their results are a valuable contribution to the literature, and ecologists should be paying more attention to limitations of the climate data they use, but as I argue in this letter, I would suggest that increasing standardization is a step backward for ecological studies.

One of the most important things to consider when assessing climatic datasets is what you will use as the truth (Daly, 2006). Terando et al. (2017) start with the assumption that the truth is air temperatures in radiation shields at a height of ~2 m. However, using the terminology of Austin (2002), air temperatures are only an indirect predictor of temperatures that are likely to be ecologically meaningful, like body, soil, or leaf temperatures. Indeed, Terando et al. (2017) note that only a third of studies with microclimatic sensors were used to record air temperatures, so the majority were observing soil, water, or other temperatures that were presumably targeted to be more biologically relevant to the species or communities of interest. We should not be discouraging researchers from observing conditions that more closely reflect those of our target species, so greater standardization of observations may appear beneficial, but actually lead to observations that are less ecologically relevant.

Many species are exposed to direct radiation to varying degrees, and so sheltering instruments may not be ideal in many circumstances. In fact, it is possible that air temperatures at 2 m are biased cold rather than microclimatic sensors biased hot when dealing with species that are exposed to direct radiation. Of course the relative biases will depend on factors like surface (or body or leaf) albedo, emissivity, moisture (latent and specific heat), etc. and so the exact biases will depend on the target species and sensors used. No methodology will be ideal for all species or applications. Researchers like Michael Kearney have produced specific microclimatic models for individual species (e.g., Kearney et al., 2008), and this is where microclimatic sensors have the greatest potential—collecting targeted data that is more relevant for species or communities than air temperatures at 2 m. In most cases, it is more appropriate to refer to microclimatic sensors as “targeted” than “ad hoc,” as the latter implies a lack of forethought which is not always the case.

The conclusion that microclimatic sensors are “highly biased” also depends on the relative size of the bias compared with other sources. For example, near‐surface air and soil temperatures on a hot summer day can be 60°C when the air temperature at 2 m is 40°C (Ashcroft & Gollan, 2012), a bias which is much larger than the 3–5°C bias attributed to radiation shielding. Similarly, Terando et al. (2017) conclude that observations of minimum temperatures are relatively unbiased, but this also overlooks the importance of sensor height. It has long been known that there can be frosts at surface level even when air temperatures at 2 m are positive (Geiger, 1950), and given that frosts can be biologically destructive, this is a crucial bias in minimum air temperatures. Therefore, in the case of both minimum and maximum temperatures, the height of observation can potentially lead to more biologically detrimental biases than those introduced by the specific radiation shield used. Similar biases could be introduced using data from a nearby standardized weather station without explicitly adjusting for cold‐air drainage, canopy cover, and other topoclimatic influences.

As an example of the dangers of relying too heavily on standardized observations, consider the proliferation of studies that have looked at species warming tolerances. Warming tolerance is defined as the difference between the physiological temperature limit of a species and the temperature in the species habitat and is generally interpreted as a buffer against climate variability or change. So, for example, Diamond et al. (2012) used WorldClim data (based on standardized observations) and compared this to physiological limits for ants. They found warming tolerances of up to 30°C; however, much of this is likely due to biases between observations at 2 m and surface or ant body temperatures rather than an actual buffer against climate change.

There are a number of promising alternatives for predicting body, surface, or leaf temperatures for use in ecological studies. Mechanistic microclimatic modeling techniques (Kearney et al., 2008) are now readily available, but often rely on assumptions or parameter estimations, and currently ignore landscape‐scale processes like cold‐air drainage. Empirical interpolation techniques (Ashcroft & Gollan, 2012) and empirically calibrated mechanistic models (Maclean, Suggitt, Wilson, Duffy, & Bennie, 2017) can capture these landscape‐scale processes but are the techniques most likely to be biased by radiation shielding issues because they require large sample sizes to accurately determine the effects of a large number of relevant climate‐forcing factors (i.e., elevation, radiation, canopy cover, cold‐air drainage, topographic exposure to winds, coastal effects, etc.), and large sample sizes inevitably require cheap sensors or excessive budgets. Infrared sensors or thermal cameras also offer an opportunity to record environmental temperatures (Scherrer & Körner, 2010). All these approaches are using advances in technology to improve the ecological realism of temperatures (create more direct predictors sensu Austin, 2002), and a push for greater standardization is moving backward in this respect.

The intent of this letter is not to deny or belittle the biases that poor radiation shielding can result in, but simply to put it into perspective of other sources of bias and error. I agree that ecologists need to pay more attention to the quality of the climate data they use, but have argued that in many cases standardized data at 2 m is not best for ecological purposes. For many species, it would be more important to move sensors closer to the ground surface than to improve radiation shields (Geiger, 1950). In the future, we will undoubtedly be predicting leaf, soil, and body temperatures by combining data from all the above approaches—standardized observations, microclimatic sensors, mechanistic models, and thermal images—to take advantage of the pros and cons of each approach. Relying on one source of data, standardized observations or any other, will limit our ability to achieve these goals and should be discouraged.

## CONFLICT OF INTEREST

None declared.
